# Variation in fat content between liver lobes and comparison with histopathological scores in dairy cows with fatty liver

**DOI:** 10.1186/s12917-017-1004-9

**Published:** 2017-04-12

**Authors:** C. Gerspach, S. Imhasly, R. Klingler, M. Hilbe, S. Hartnack, M. Ruetten

**Affiliations:** 1 Department of Farm Animals, Vetsuisse Faculty, University of Zurich, Winterthurerstrasse 260, Zurich, Switzerland; 2 Institute of Pharmacology and Toxicology, Vetsuisse Faculty, University of Zurich, Winterthurerstrasse 260, 8057 Zurich, Switzerland; 3 Institute of Veterinary Pathology, Vetsuisse Faculty, University of Zurich, Winterthurerstrasse 268, Zurich, Switzerland; 4 Section of Epidemiology, Vetsuisse Faculty, University of Zurich, Winterthurerstrasse 270, Zurich, Switzerland

## Abstract

**Background:**

The assessment of a liver biopsy remains the gold standard for diagnosing and staging fatty liver in dairy cows, which is often necessary for diagnostic and research purposes. Accuracy of the diagnosis relays on the quality of the biopsy, the assumed representativeness of a small tissue sample for a disease process throughout the liver and accurate human evaluation of histologic specimens. The objective of the present study was to assess the distribution of triacylglycerol (TAG) infiltration throughout the parenchyma of livers with different degrees of fatty liver in dairy cows. In addition, histopathological scores from the corresponding specimens were compared to a quantitative measurement of TAG, as well as the agreement between two observers.

**Methods:**

Thirty livers with different degrees of lipid infiltration were selected and 10 different locations throughout the liver were assessed. The TAG content was measured enzymatically, calculated in % or mg/g wet weight, and assigned to a scoring system. Corresponding tissue specimens were stained with hematoxylin-eosin (H&E) and Oil red O (ORO) for histopathological evaluation, using a scoring system.

**Results:**

The difference in TAG content between any locations was less than 2%. Based on the scoring system the TAG concentration was even distributed in 79.3% of the livers. Based on kappa statistics the agreement between two pathologists and staining technique in scoring histological specimens was moderate to fair.

**Conclusions:**

Overall the distribution of TAG throughout the liver and the accuracy of human evaluation of liver biopsies may lead to acceptable diagnoses for clinical purposes. Within the liver lobules a common pattern of lipid distribution depending on severity could be observed. For the staging of lipid infiltration for research projects, some degree of variation needs to be considered.

## Background

Fatty liver is a metabolic disease occurring during the transition period in dairy cattle. Diagnosis and staging of fatty liver is often necessary for diagnostic and research purposes. Lipid infiltration can reach from a physiological state, in response to negative energy balance in early lactation, to a progressed state, with altered liver function and clinical disease. The clinical symptoms are unspecific [1]. Therefore, the main reasons to evaluate liver biopsies are establishing a morphological diagnosis and to assess the severity of disease. The lipid content of liver tissue can be evaluated by biochemically, histologically or by specific gravity [1]. For quantitative determination of triacylglycerol (TAG) or total lipid content, the TAG content of liver tissue can be quantitatively measured enzymatically using an automated analyzer [2]. The histologic evaluation of liver tissue is a technique, routinely used for clinical but also for research purposes.

Two issues influencing an accurate diagnosis have been reported in different species. First the tissue sample should be representative for the disease which depends on the method of collection, size and number of percutaneous biopsies [3–7]. In addition, the assessment of a biopsy is only successful in diffuse hepatopathies. A percutaneous liver biopsy is always taken from the right lobe and represents 0.002 to 0.1% of the whole liver parenchyma, depending on the size of the biopsy needle [8]. Fatty liver in cattle is reported to be diffuse [8]. However, in beef bulls concentrations of cholesterol and phospholipids were higher in the intermediate lobe, compared to the right and left lobes [9]. Local foci of fatty degeneration in livers of dairy cows have been reported, which are due to tension and resulting in ischemia at the insertion of a serosal attachment (tension lipidosis) [10]. Focal fatty liver with unknown etiology has been diagnosed in a heifer [11]. In dogs and cats liver lesions of different etiology were partially uneven distributed in disorders usually regarded as diffuse [5].

Second, histological evaluation of liver tissue is prone to bias [12–15]. In a study investigating human evaluation of liver steatosis in people revealed a high level of variability between human observation and standardized computer measurement. At the same time a disagreement of up to 37% between observers was determined [13].

Despite several attempts to find and develop alternative and less invasive diagnostic methods in the past, the evaluation of a liver biopsy remains the current gold standard in diagnosing and investigating fatty liver in dairy cows.

The goal of our study was to investigate the distribution of TAG infiltration throughout livers with different stages of fatty liver, by comparing biopsies from 10 different localizations. In addition, we compared histopathological scores to a quantitative measurement of triacylglycerol in liver tissue and the agreement between two observers, using two different 2 embedding methods.

## Methods

### Collection of liver tissue

**Fig. 1:**
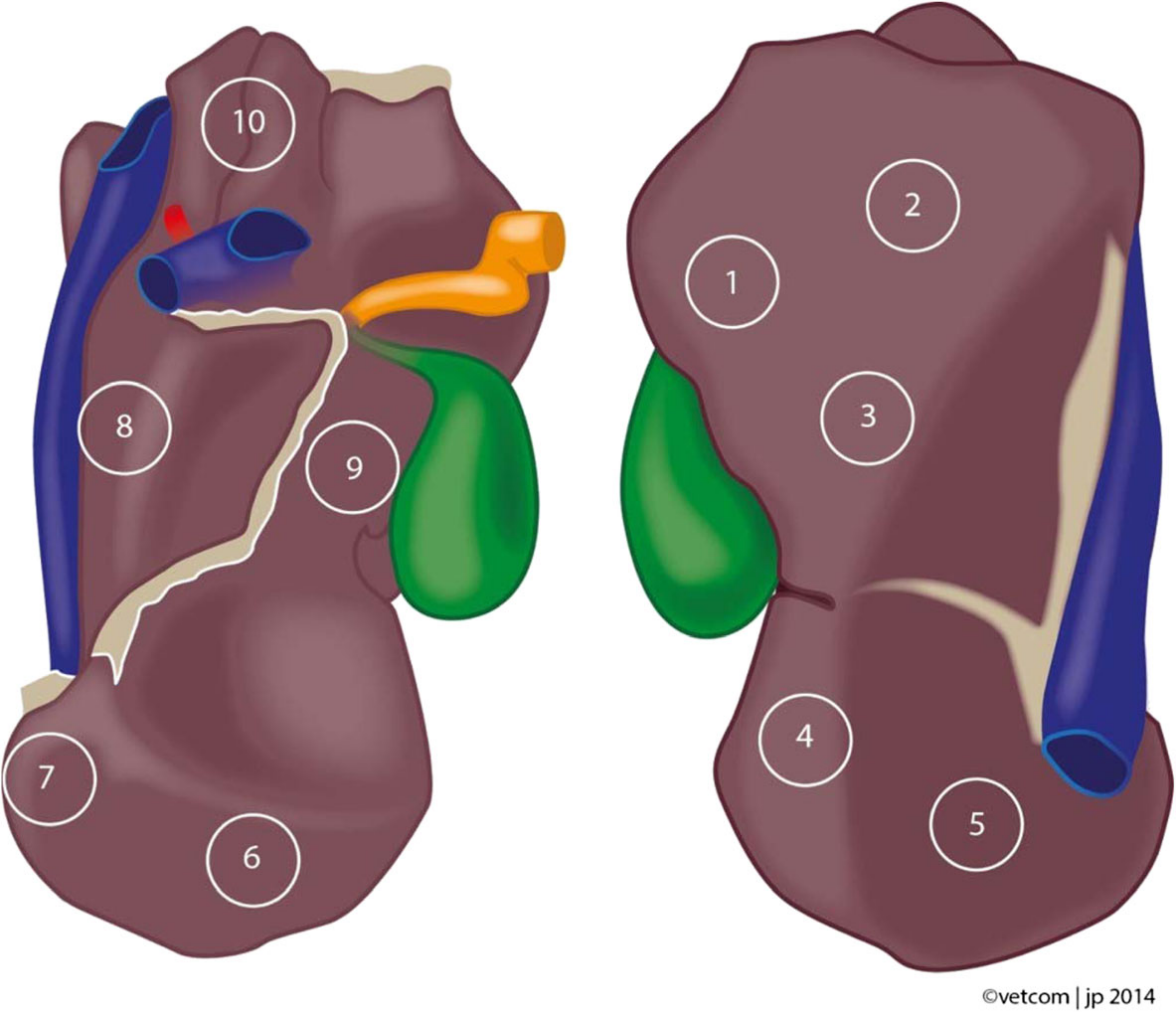
Liver with 10 locations for tissue sampling (1–3 = right lobe; 4–5 = left lobe, diaphragmatic surface; 6–8 = left lobe, visceral surface; 9 = quadrate lobe; 10 = caudate lobe)

Wedge biopsy specimens of livers were taken at an abattoir. The selection was focused on livers with potential lipid infiltration. Liver samples were excised immediately after slaughter from 10 different localizations of each liver, using a scalpel (Fig. 1). The tissue samples from the corresponding localizations were placed into appropriate tubes for histopathologic examination and measurement of TAG.

### Histopathology

**Fig. 2:**
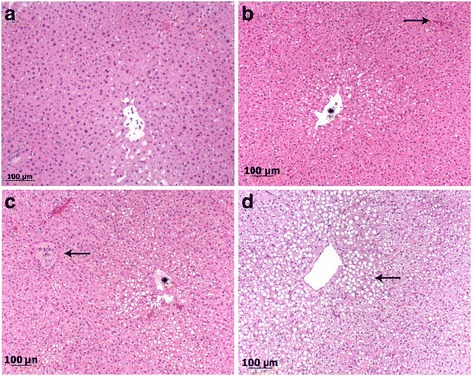
Bright field images of different histological grades of fatty liver degeneration. **a** Grade 0: one lobule with a central vein of a normal liver, no lipid vacuoles in the cytoplasm. **b** Grade 1: small rim of hepatocytes with clear demarcated fatty vacuoles round a central vein (star). The portal field marking the edge of the liver lobule is marked with an arrow. **c** Grade 2: half of the hepatocytes show fatty vacuoles, central vein (*star*) and portal field (*arrows*) are marked. **d** Grade 3: all hepatocytes of the lobule show lipid vacuoles. The vacuoles in hepatocytes around the central vein are bigger (*arrow*)

**Fig. 3:**
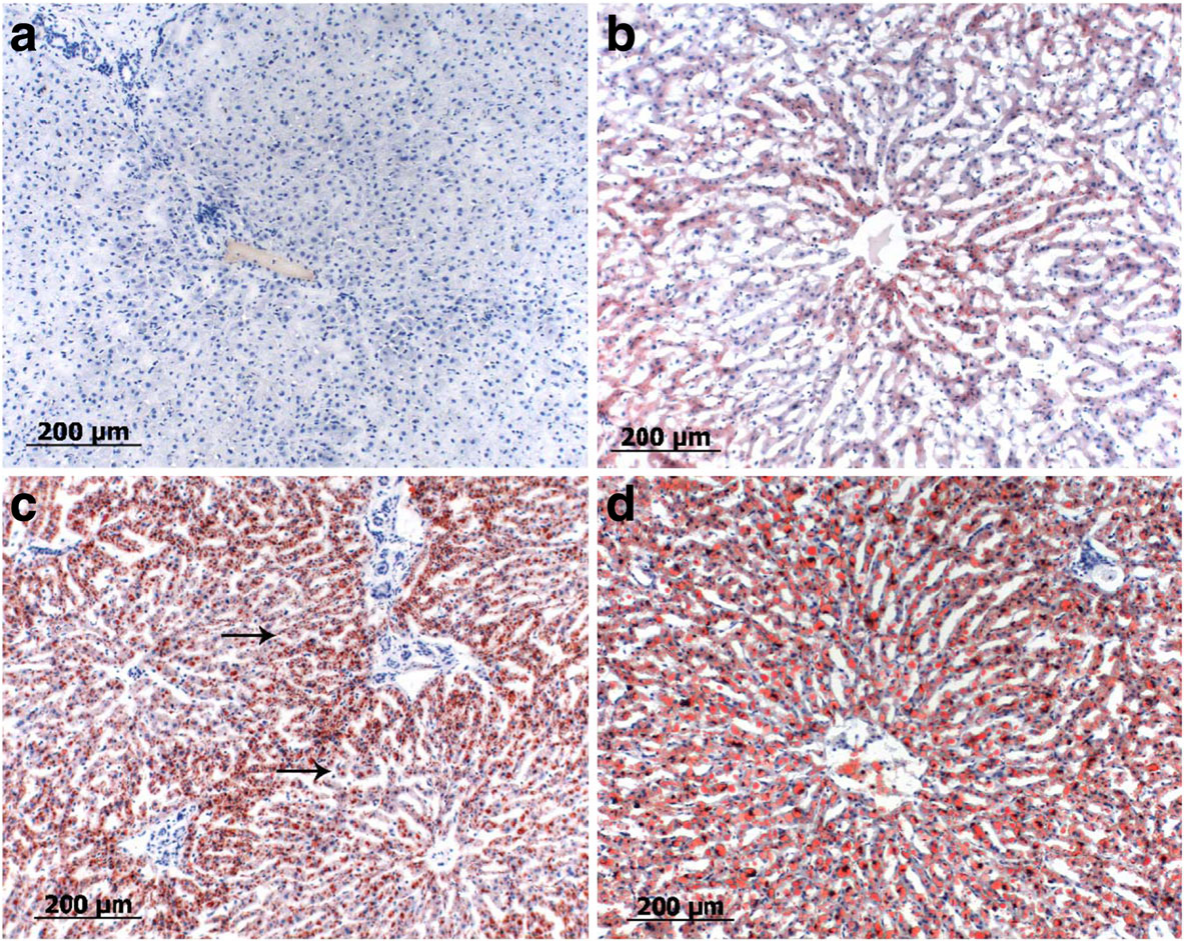
Bright field images ORO staining of different histological grades of fatty liver degeneration. **a** Grad 0: normal liver, no fat deposition visible. **b** Grade 1: Positive red signal intracytoplasmatically in hepatocytes around central vein. **c** Grade 2: half of hepatocytes of a liver lobule show clear red stained fatty vacuoles. The edge to an area with only finely red stained lipid droplets is marked with arrows. **d** Grade 3: entire liver lobule shows red stained, clear demarcated fatty vacuoles in hepatocytes

For histopathology, tissue samples were collected with a size of 1 cm × 1 cm × 1 cm approximately 1 cm under the surface and fixed in 10% buffered formalin, and small samples were snap frozen in liquid nitrogen. The samples fixed in formalin were dehydrated by an ascending alcohol series ending in xylol and finally embedded in paraffin. The specimens were sectioned at a thickness of 2–3 μm and sections were stained with hematoxylin-eosin (H&E), periodic acid-Schiff stain (PAS) or Oil red O (ORO). The snap frozen samples were cut directly in 3–4 μm sections and stained with PAS and ORO without any prior fixation (alcohol) applied. The histological lesions were staged, according to a scoring system, into 4 categories: 0 = no abnormalities; 1 = moderate fatty liver (only cells from one zone are affected; periportal, midzonar or zentrolobular); 2 = severe fatty liver (the periportal and midzonal or midzonal to centrolobular areas are affected); 3 = very severe fatty liver (all three zones are affected, including the Kupffer cells) for both, H&E (Fig. 2) and ORO (Fig. 3). Respectively, the same grading scale was used for intracellular glycogen depositions. All specimens were evaluated by two board-certified pathologists (MR, MH) with a high level of experience, blinded to liver localizations and animal identification.

The intracellular vacuoles were classified according to morphology either as fatty vacuoles or glycogen deposition. Clearly demarcated vacuoles with or without displacement of the nucleus, was classified as fatty vacuole. Foamy cytoplasm and a centrally placed nucleus within slightly swollen hepatocytes was considered as glycogen deposition. These findings were verified by using either PAS (glycogen) or ORO (fat) staining.

### Liver tissue triglyceride measurement

Small samples of liver tissue were placed in 2 ml Eppendorf Safe-Lock tubes (Eppendorf, Germany) immediately snap frozen in liquid nitrogen, and stored at −85 °C for later determination of TAG content. After thawing, analysis was performed by tissue saponification in ethanolic KOH as described [16]. In short, approximately 100 mg of tissue samples were transferred to new pre-weighed 2 ml tubes, after removing non-parenchymatos tissue, and the exact weight recorded. By adding 350 μl of ethanolic KOH (2 parts EtOH: 1 part 30% KOH) incubation was performed overnight, at 55 °C in a thermos shaker until tissue was completely digested. 650 μl water:EtOH (1:1) were added and centrifuged at a speed of 16′000 G for 5 min. Supernatant was transferred in a new tube and 200 μl water:EtOH (1:1) were added and vortexed. 200 μl of the mixture were transferred in a new tube and 215 μl 1 M MgCl_2_ were added and vortexed and left on ice for 10 min. Finally, the tubes were centrifuged at 16′000 G for 5 min and the supernatant was transferred in a new tube and stored at −20 °C until measurement.

The TAG content was measured enzymatically with the free glycerol reagent (Sigma-Aldrich). As triolein equivalent (CTE) standard curve were produced by glycerol standard (Sigma-Aldrich). In a 96 well plate, 6 μl of the sample were added to 200 μl of the free glycerol reagent, incubated for 15 min at room temperature and read at 540 nm. The blank were subtracted from the samples/standards and the concentration was calculated comparing to the standard curve. Each sample was measured in triplicates.

The results were also calculated in mg/g wet liver and assigned to a scoring system as used by Haudum et al. (2011): 0 = ≤ 50 mg/g (mild); 1 = ≥ 51–100 mg/g (moderate); 2 = ≥ 100–150 mg/g (severe); 3 = ≥ 150 mg/g (very severe).

### Data analysis

Descriptive statistics was performed using GraphPad Prism (Prism 6.05, GraphPad Software, Inc., La Jolla, CA, USA). The agreement between two observers, assessing the same samples, was calculated using the κ test (unweighted kappa, www.vassarstats.net). Values of 0–0.2 represented a slight agreement, 0.21–0.4 a fair, 0.41–0.60 a moderate, 0.61–0.8 substantial and >0.8 almost perfect agreement [17].

Linear mixed effects models were utilized to assess if TAG concentration differed significantly between locations.

Generalized linear mixed effect models were used to assess if disagreement between the two observers could be explained by the TAG concentration. Animal was considered as random effect, TAG concentration and localisation as fixed effects. Model selection was based on AIC (Akaike’s information criterion) with lower values indicating a better model fit. The analysis was performed with the software R (R Core Team 2015) and the package lme4 [18].

The intra-assay coefficient of variation (CV) expressed as a percent value was calculated as CV = within-assay standard deviation/mean × 100. The intra-assay CV was 4.8%.

## Results

### Animals

A total of 300 liver samples from 30 animals were examined for histologic findings and TAG content. The cows were between 2.5 and 9 years old and 4 to 500 days post-partum. They belonged to the breeds Red Holstein (*n* = 11), Brown Swiss (*n* = 6), Holstein Frisian (*n* = 6), and Montbeliard (*n* = 1). In 3 animals breed, age, and number of days in milk could not be determined.

### Pathological assessment of liver tissue

**Fig. 4:**
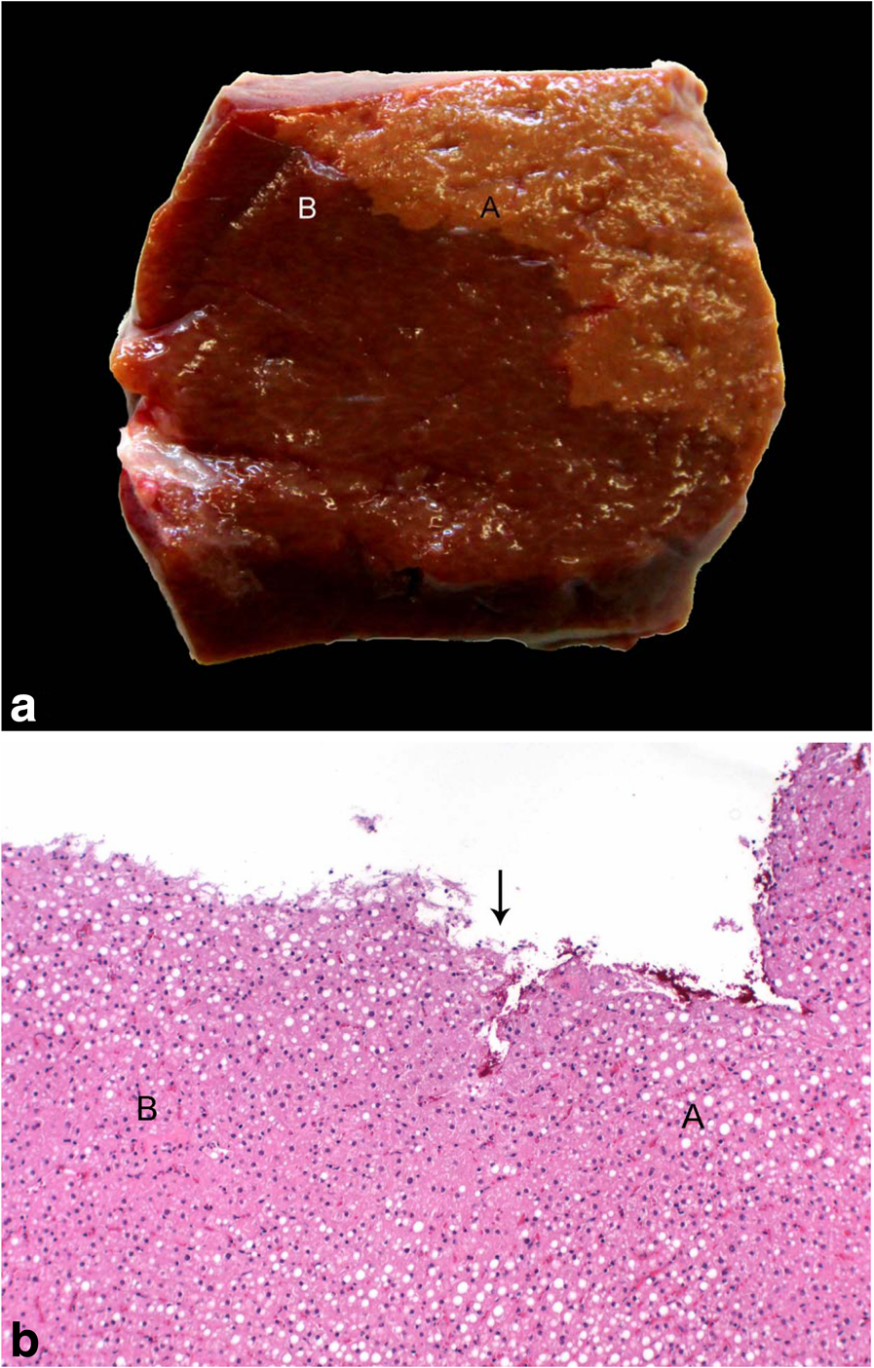
(a) Liver tissue with heterogeneous distribution of lipid infiltration (A: normal liver, B: area with suspected fatty degeneration), (b) H&E stained tissue from corresponding specimen (A: normal liver, B: area with suspected fatty degeneration), arrow: border between macroscopically different appearance; no difference of fat deposition could be found within the hepatocytes

Macroscopically 29 of 30 livers had a homogenous appearance with a slightly brighter brownish coloration than normal liver tissue. One liver showed few, randomly distributed clearly demarcated, light brown to yellowish foci that were not associated to any ligament insertion site (Fig. 4a). The histopathological examination did not reveal obvious differences in the degree of lipid infiltration in that liver (Fig. 4b).

Based on the average score of 10 different localizations from each liver, the histological assessment revealed an overall diagnosis of fatty liver in up to 25/30 livers, depending on the pathologist and staining technique (Table 1). No concomitant liver lesions ore evidence of inflammation were detected. In specimens stained with HE, the scores were evenly distributed throughout all 10 localizations in agreement of both observers, in 22/30 livers (73.3%). In 4/30 (13.3%) livers the observers disagreed in their grading.

**Table 1:** Overall scores for 30 livers. HE, H&E stain; ORO, Oil Red Stain; MR and MH, pathologists

	H&E		ORO	
liver	MR	MH	MR	MH
1	0	0	0	1
2	0	0	0	1
3	0	0	0	1
4	3	3	3	3
5	0	0	0	0
6	0	0	0	0
7	0	0	0	0
8	0	0	0	0
9	0	0	0	1
10	0	0	0	0
11	0	0	0	2
12	0	0	0	1
13	1	2	2	2
14	0	0	0	1
15	1	2	1	3
16	2	3	3	3
17	0	1	1	3
18	0	1	1	1
19	3	3	3	3
20	1	2	1	3
21	1	1	2	2
22	0	0	0	1
23	1	1	1	2
24	0	0	0	1
25	1	2	2	2
26	3	3	3	3
27	3	3	3	3
28	0	1	0	1
29	2	2	3	3
30	2	2	3	3

*κ* kappa reliability test, *CI* confidence interval

The scores were even distributed in 17/30 (56.7%) livers stained with ORO throughout all 10 localizations and were in agreement of both observers. In 1/30 (3.3%) livers, findings were not distributed diffuse throughout all localizations, observed by both pathologists. In 11/30 (36.7%) livers, there was a disagreement between observers. Overall the agreement between the two observers was fair to moderate in evaluating samples stained with H&E and fair in samples stained with ORO (Table 2). The agreement between H&E and ORO was fair to moderate (Table 3). Both observers gave slightly higher scores when evaluating ORO stained samples.

**Table 2:** Agreement between observers for each localization and overall (whole livers)

Liver	H&E		ORO	
	κ	95% CI	κ	95% CI
1	0.70	0.49–0.90	0.29	0.08–0.50
2	0.60	0.38–0.81	0.29	0.08–0.50
3	0.54	0.31–0.76	0.37	0.15–0.59
4	0.56	0.33–0.78	0.30	0.09–0.51
5	0.62	0.40–0.85	0.29	0.07–0.50
6	0.64	0.41–0.86	0.29	0.08–0.51
7	0.45	0.22–0.68	0.21	0.01–0.41
8	0.54	0.32–0.76	0.22	0.01–0.42
9	0.49	0.27–0.71	0.23	0.03–0.44
10	0.74	0.54–0.93	0.29	0.08–0.49
overall	0.58	0.51–0.65	0.28	0.21–0.35

**Table 3:** Agreement between H&E and ORO for each localization and overall (whole liver)

Liver	MR		MH	
	κ	95% CI	κ	95% CI
1	0.64	0.40–0.87	0.38	0.14–0.62
2	0.59	0.34–0.83	0.43	0.20–0.66
3	0.53	0.28–0.79	0.48	0.25–0.71
4	0.47	0.20–0.74	0.20	0.00–0.42
5	0.57	0.31–0.82	0.34	0.12–0.58
6	0.57	0.32–0.83	0.35	0.11–0.58
7	0.57	0.32–0.83	0.34	0.11–0.58
8	0.58	0.34–0.83	0.36	0.13–0.59
9	0.54	0.29–0.79	0.52	0.29–0.74
10	0.53	0.28–0.79	0.29	0.06–0.51
overall	0.56	0.48–0.64	0.34	0.29–0.44

*κ* kappa reliability test, *CI* confidence interval

The evaluation of H&E stained specimens revealed 76/300 (25.3%) samples being diagnosed with different scores. Of those only 2/300 (0.7%) differed by 2 scores. In liver tissue stained with ORO, there was a disagreement in 163/300 (54%) specimens. In 35/300 (11.7%) specimens there was a difference of 2 scores.

**Fig. 5:**
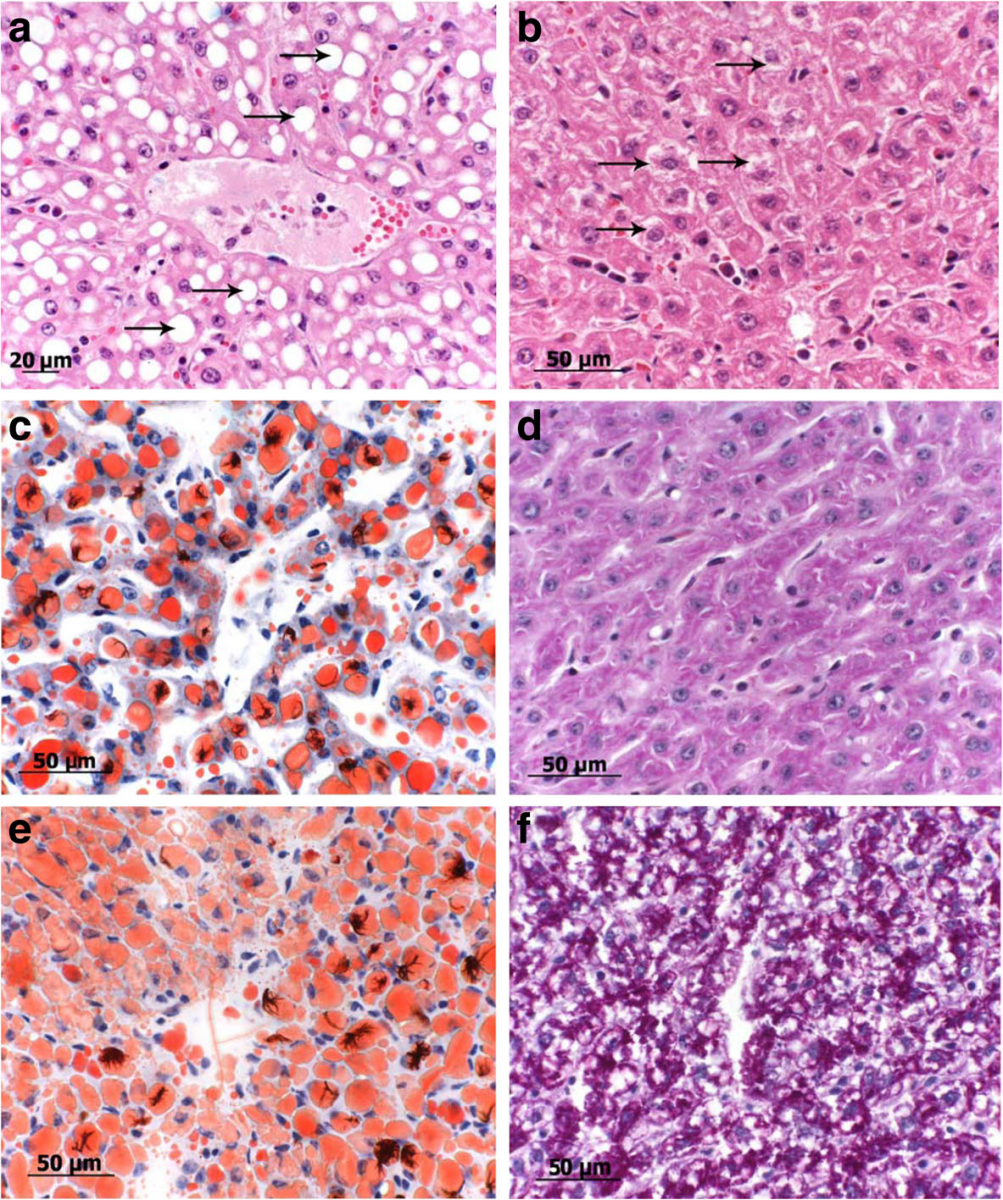
Bright field images, comparison different morphology of fatty vacuoles and glycogen depositions of formalin fixed and snap frozen liver samples, H&E, PAS and ORO staining. **a** large, clear demarcated fatty vacuoles intracytoplasmatically in hepatocytes displacing the nucleus excentrically. **b** Intracytoplasmatical glycogen deposition (arrows) in swollen hepatocytes showing a foamy brightened cytoplasm. **c** ORO staining of formalin fixed tissue sample, showing clear red stained fatty vacuoles diffusely distributed in the liver lobule. **d** PAS staining of glycogen deposition of formalin fixed tissue samples. **e** ORO staining of snap frozen liver sample, diffuse red coloration of hepatocytes, unclear cell borders, sinusoids indistinct and difficult to localize. Distribution of fatty vacuoles similar to formalin fixed tissue. **f** PAS staining of snap frozen liver samples, diffuse pinkish coloration of hepatocytes, unclear cell borders, sinusoids indistinct and difficult to localize. Distribution of glycogen depositions similar to formalin fixed tissue

The distribution of intracellular glycogen was negatively correlated with the distribution of fatty depositions (data not shown). There were no significant differences found between formalin fixed and snap frozen samples. However, the morphology of the cell borders, vacuoles and nucleus of hepatocytes in the slightly thicker, not fixed sections were more difficult to assess. The intensity and distribution of coloration of the special staining was comparable in either method (Fig. 5).

### Biochemical analysis of hepatic TAG content

**Fig. 6:**
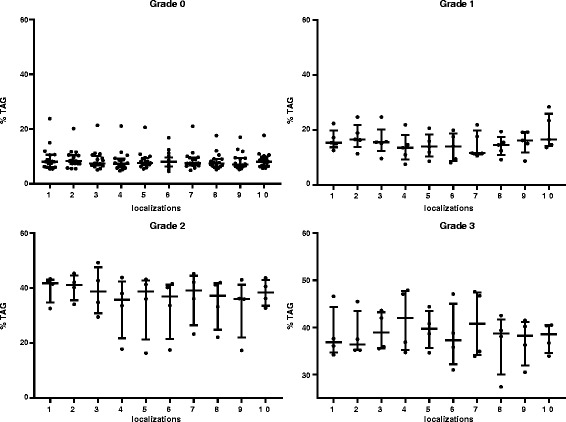
TAG content (% wet weight) of 10 localizations from 29 livers with degree 0 to 3

The percentage of TAG from wet liver of 10 locations from 29 livers was measured (Fig. 6). Tissue from liver no. 25 was not available for TAG measurement. Based on a linear mixed effect model approach, location was found to be significantly associated with TAG concentration (*p* = 0.003), albeit the difference between any two localizations was smaller than 2%. Table 4 demonstrates the comparison of localization 1, which is usually the side for percutaneous biopsy, with localizations 2 to 10. Localizations 4,5,6,8, and 9 had significantly different TAG concentrations, all of them being less than 2%. Scoring of 290 liver tissue samples revealed 124 specimens with moderate, 49 with severe, and 117 specimens with very severe fatty liver. Based on the scoring system the TAG concentration was even distributed in 79.3% of the livers. In 4 of 29 livers, the scores differed by 1 and in 2 livers by 2 scores. Mean, standard deviation and range of the measured values of each liver are shown in Table 5. The highest variation in TAG content within one liver was 25% (liver no. 13). Since the TAG content was >150 mg/g in all 10 locations, the scores were equally throughout the liver. The same liver was diagnosed with marked variation, based on histopathological evaluation. The variation between locations was significantly higher in livers diagnosed with scores 2 or 3 compared to livers with a score of 1.

**Table 4:** Comparison of the localizations 2–10 to localization 1 (baseline; usual side for percutaneous biopsy) using a linear mixed effect model approach

Localization	Value	Std. Error	*p*-value
1 (intercept)	18.8	2.5	0.0000
2	0.1	0.7	0.8892
3	−0.4	0.7	0.5607
4	−1.5	0.7	0.0279
5	−1.4	0.7	0.0389
6	−2.0	0.7	0.0048
7	−0.8	0.7	0.2532
8	−1.9	0.7	0.0056
9	−1.8	0.7	0.0090
10	−0.2	0.7	0.7654

**Table 5:** TAG content (% wet weight) in 29 livers. Mean, standard deviation, and range of measured values for 29 livers

Liver	Mean	Std Dev.	Range
1	6.6	0.67	5.5–7.7
2	7.3	0.93	5.8–8.3
3	7.6	0.72	6.8–9.3
4	33.4	2.88	27.4–36.9
5	5.4	0.37	4.9–6.2
6	5.5	0.62	4.5–6.4
7	6.5	0.41	5.9–7.3
8	6.2	0.55	5.5–7.4
9	11.3	1.0	9.1–12.4
10	10.0	0.83	8.9–11.2
11	11.3	1.53	10.0–15.0
12	7.7	0.54	6.9–8.5
13	26.9	9.92	16.4–41.5
14	9.1	0.75	8.2–10.7
15	21.8	2.05	19.1–24.7
16	34.3	1.37	32.6–36.1
17	19.72	2.32	16.8–23.8
18	7.93	0.91	6.6–9.7
19	36.4	1.83	34.7–40.8
20	16.0	1.13	14.5–17.6
21	11.0	3.48	7.5–19.2
22	8.9	0.57	8.1–10.3
23	14.5	5.91	8.7–28.4
24	7.2	0.44	6.5–8.0
25	-	-	-
26	44.7	2.64	40.5–47.9
27	40.1	3.46	37.5–47.1
28	14.5	1.36	11.6–16.5
29	42.9	2.51	40.3–49.3
30	41.9	2.95	36.2–45.4

**Fig. 7:**
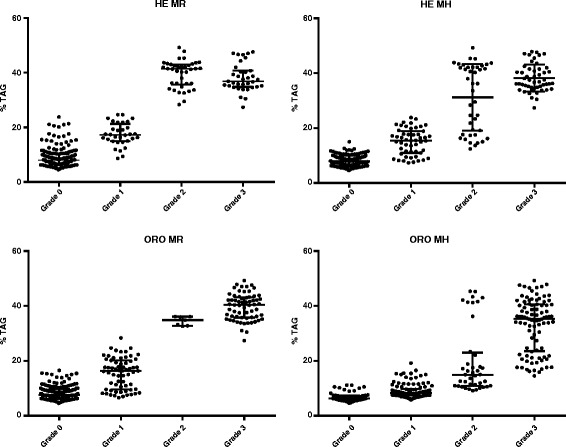
TAG content (% wet weight) of 29 livers, according to staining technique (H&E, ORO) and pathologists (MR, MH)

In Table 6 the mean TAG content is listed for each grade of fatty liver, using H&E and ORO stains (also Fig. 7).

**Table 6:** Mean and SD of TAG content for each score. HE, H&E stain; ORO, Oil Red Stain; MR and MH, pathologists

	Liver TAG (% wet weight)			
	MR		MH	
	HE	ORO	HE	ORO
Grade 0	9.0 (± 3.7)	8.3 (± 2.6)	7.9 (± 2.0)	6.7 (± 1.7)
Grade 1	17.5 (± 4.2)	15.5 (± 5.6)	15.0 (± 4.7)	9.1 (± 2.7)
Grade 2	39.6 (± 5.0)	34.5 (± 1.6)	31.2 (± 12.7)	20.1 (± 12.6)
Grade 3	38.2 (± 5.1)	39.7 (± 4.8)	38.2 (± 4.9)	33.2 (±9.6)

The TAG concentration did not influence the agreement between observers, which was tested using a generalized linear mixed effect model.

## Discussion

The examination of a liver biopsy remains the gold standard for diagnosing and staging fatty liver. Important factors affect an appropriate diagnosis of liver biopsies, including the quality of the biopsy, the small tissue sample representing the lesion of the whole organ, and the interpretation of histological findings. Accurate assessment of the degree of fatty liver is a key requirement for staging, required for research purposes.

The results of this study demonstrate minor variation in the distribution of lipids in 10 different locations within the liver of dairy cows. The TAG content was up to 25% different within one liver. The difference in TAG content between any location was less than 2%. Therefore, the TAG content of a liver biopsy taken either at the right lobe (percutaneous) or at the quadrate lobe (during laparotomy) differs less than 2% from other locations throughout the liver.

In humans, lesions of nonalcoholic fatty liver disease are unevenly distributed [7]. However, caution must be used in extrapolating results in humans to cows. In the study of Ratziu (2005) the steatosis grade displayed substantial agreement between 2 biopsies, whereas inflammation and fibrosis did not. In cattle, hepatic lipidosis is a reversible process and inflammation and fibrosis are usually not seen [1, 19]. Inflammatory cells or fibroblasts could not be detected on our samples.

**Fig. 8:**
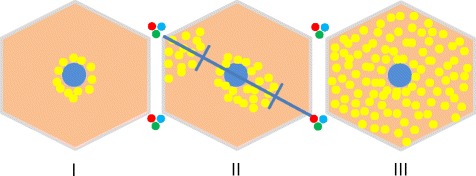
Distribution of lipid infiltration within hepatic lobules, according to the severity of fatty liver. I = grade 1 (infiltration in one zone, around central vein); II = grade 2 (infiltration periportal to midzonal or zentrolobular to midzonal); III = grade 3 (infiltration panlobular)

One common feature with human NAFLD was the distribution of lipid infiltration within the hepatic lobules (Fig. 8). In mild cases we could observe infiltrations close to the central vein. With increasing severity the lipid infiltration extended to all zones. In two cases with mild fatty liver, infiltration could be found more periportal (Table 7). Because of the cross-sectional nature of this study, we could not demonstrate the changes in lipid infiltration over time.

**Table 7:** Number of whole livers with different grades in association to the localization of lipid infiltration within hepatic lobules

	Periportal	Zentrolobular	Zentro-panlobular	Panlobular
Grade 1	2	4	0	0
Grade 2	0	0	4	0
Grade 3	0	0	1	5

Based on histopathologic scores the pathologists agreed with an even distribution of lipid infiltration in 73.3% of the livers. The histopathological evaluation of the liver tissue revealed some disagreement between the pathologists and between staining technique. Overall ORO staining revealed slightly higher scores compared to H&E staining. Differences in histopathologic interpretation between observers is well known [3]. Human evaluation of biopsies is prone to bias [13], but to date there is also no consensus for an objective grading and staging system for the histological evaluation of fatty liver in cattle. Therefore, there might be differences in the prevalence of hepatic changes between studies [3]. In addition, without accurate and widely accepted methods it is difficult to compare findings related to TAG content in liver tissue between studies [20].

In the literature, different scores are used for lipid infiltration in fatty liver [21, 22]. Therefore, the comparison between scores based on histopathology and measured TAG is difficult.

The content of TAG was significantly higher in livers diagnosed with fatty liver compared to livers with a histopathologic score of 0. However, there was no significant difference in TAG content between the histopathologic scores 2 and 3. Best agreement with the quantitative TAG content could be achieved with ORO staining. This is in agreement with another study, performed with human and rat livers, which revealed good agreement between histopathologists based on H&E assessment but an inaccuracy compared to the true hepatic TAG concentrations [15]. The ORO stain was more accurate, because ORO identifies mainly TAG and less phospholipids and cholesterol [15]. A steriological analysis of fatty livers revealed a highly significant correlation with the hepatic TAG content in cattle [8]. Also Collins et al. revealed a high correlation between TAG content and volume fraction of fat droplets determined stereologically [23]. In our study we used a more practical technique, which is routinely used for the diagnosis of fatty liver and H&E stained specimens are often available for retrospective evaluation of cases.

In addition, one observer appeared to be stricter in scoring the specimens. A disagreement by one grade might also be due to the fact, that a categorical scoring system is used for a continuous disease process.

Formalin fixing and routine processing of the tissue samples can be an accurate method for histological assessment and grading. Even with the loss of fat or glycogen content a grading can be accomplished. The morphological appearance of cytoplasmic vacuoles, well demarcated versus foamy depositions/appearance, can be distinct enough to differentiate between fatty and glycogen depositions in H&E staining. However, special staining techniques improve the accuracy, especially when droplet size is quantified, although not being practical [15].

We suspected focal lipid infiltration macroscopically in one liver. However, the histopathological examination did not reveal obvious differences in the degree of lipid infiltration between the corresponding tissue samples. Focal fatty liver in cattle was rarely reported in the literature. Mohamed et al. [11] diagnosed focal fatty liver by ultrasonography in a heifer. In a study population of 106 Holstein cattle focal fatty liver was observed in 24% of the cows [10]. Focal lipid infiltration occurs adjacent to the insertion of serosal attachments [24] and is not related to the pathogenesis of fatty liver in cattle. In contrast to our results Raoofi et al. [10] and Mohamed et al. [11] observed a higher amount of fat vacuoles compared to the surrounding tissue.

Overall the distribution of TAG throughout the liver and the accuracy of human evaluation of liver biopsies may lead to acceptable diagnoses for clinical purposes. For the staging of lipid infiltration for research projects, some degree of variation needs to be considered.

A consensus on histopathologic diagnosing and staging of hepatic lipid infiltration should be established. In our study, we used a scoring system including the observation of lipid infiltration in the different lobular zones in order to divide into 4 categories (grade 0 to 3). We observed that mild infiltration was mainly located centrolobular and with increasing severity the distribution was panlobular. Although experienced pathologists evaluated the tissue, the assignment to the lobular zones may be somewhat subjective. An objective measurement of the lobules may have increased the accuracy. In addition, we used samples that were obtained at one point in time for every individual cow. In order to establish a reliable protocol for histopathological diagnostics, the development of fat infiltration over time should also be investigated.

## Conclusion

In the present study, the difference in TAG content between any locations was less than 2%.

Therefore, the TAG content of a liver biopsy taken at any side differs less than 2% from other locations throughout the liver. Based on the scoring system the TAG concentration was even distributed in 79.3% of the livers. The agreement between two pathologists in scoring histological specimens was moderate to fair. Overall the distribution of TAG throughout the liver and the accuracy of human evaluation of liver biopsies may lead to acceptable diagnoses for clinical purposes. For the staging of lipid infiltration for research projects, some degree of variation needs to be considered. A consensus on histopathologic diagnosing and staging of hepatic lipid infiltration should be established.

## Abbreviations

H&E: Hematoxylin-eosin
ORO: Oil red O
PAS: Periodic acid-Schiff stain
TAG: Qtriacylglycerol
